# Effects of transient unilateral functional brain disruption on global neural network status in rats: a methods paper

**DOI:** 10.3389/fnsys.2014.00040

**Published:** 2014-03-21

**Authors:** Willem M. Otte, Kajo van der Marel, Kees P. Braun, Rick M. Dijkhuizen

**Affiliations:** ^1^Biomedical MR Imaging and Spectroscopy Group, Image Sciences Institute, University Medical Center UtrechtUtrecht, Netherlands; ^2^Department of Pediatric Neurology, Brain Center Rudolf Magnus, University Medical Center UtrechtUtrecht, Netherlands; ^3^Molecular Imaging Program at Stanford, Stanford UniversityStanford, CA, USA

**Keywords:** rat brain, resting-state fMRI, transient brain impairment, neural network, rich-club effect

## Abstract

Permanent focal brain damage can have critical effects on the function of nearby as well as remote brain regions. However, the effects of transient disturbances on global brain function are largely unknown. Our goal was to develop an experimental *in vivo* model to map the impact of transient functional brain impairment on large-scale neural networks in the absence of structural damage. We describe a new rat model of transient functional hemispheric disruption using unilateral focal anesthesia by intracarotid pentobarbital injection. The brain's functional status was assessed with resting-state fMRI (rs-fMRI) and electroencephalography (EEG). We performed network analysis to identify and quantify highly connected network hubs, i.e., “rich-club organization,” in pre- and postbarbital functional networks. Perfusion MRI data demonstrated that the catheterized carotid artery predominantly supplied the ipsilateral hemisphere, allowing for selective hemispheric brain silencing. The prebarbital baseline network displayed strong functional connectivity (FC) within and between hemispheres. Following pentobarbital injection, the disrupted hemisphere revealed increased intrahemispheric FC with concomitant decrease of interhemispheric connectivity. The bilateral functional network was characterized by a strong positive rich-club effect, which was not affected by ipsilateral disruption. Nevertheless, the rich-club value was significantly decreased in the ipsilateral hemisphere and to a lesser extent contralaterally. Loss of interhemispheric EEG synchronization supported the rs-fMRI findings. Our data support the concept that densely connected rich-club regions play a central role in global brain communication, and show that network hub configurations can be significantly affected by focal temporary functional hemispheric disruption without structural neuronal damage. Further studies with this rat model will provide essential additional insights into network reorganization patterns in response to transient functional brain disruption.

## Introduction

Focal brain damage, such as after ischemic stroke or in epilepsy, not only affects local neuronal networks, but can also have significant effects on the function of remote brain regions. For instance, constrained behavioral output (Carter et al., 2010) and cognitive comorbidities (Tatemichi et al., 1994; Motamedi and Meador, 2003) after stroke and epilepsy have partly been attributed to functional disturbances of distant brain regions that were not primary affected. In addition, spontaneous partial recovery of sensorimotor and behavior output in the weeks and months following stroke may be explained by functional plasticity of remote and unaffected brain regions (Langhorne et al., 2009; Stinear, 2010). As brain functions are believed to emerge from interacting regions within large-scale neural networks, the brain's intrinsic capacity to compensate for focal injury is linked to reorganization of surviving functional networks at a global level (Grefkes and Fink, 2011).

A sophisticated set of measures, using the concept of graph theory, has been used to describe alterations in functional network organization following focal brain damage (Rubinov and Sporns, 2010), with promising opportunities for applications in clinical settings (Fornito et al., 2013). Obviously, changes in functional networks are strongly dependent on the structural status of neuronal connections that link brain regions. Recent studies have reported a high similarity between functional network connectivity and underlying structural neuronal connections (Achard et al., 2008; Honey et al., 2009). However, it is largely unknown how functional networks reorganize in response to transient focal disturbances without structural damage. Characterizing the impact of transient localized functional brain disruptions on large-scale neural networks can provide important insights into a network's resilience, which might lead to improved understanding of underlying causes of comorbidities in diseases with localized brain damage, such as focal epilepsy and ischemic stroke.

Global neural networks are known to have a hierarchical structure with the existence of a number of well-connected and highly central hub regions (Hagmann et al., 2008; Bullmore and Sporns, 2012) that form a so-called “rich-club,” which reflects a network's capacity to segregate and integrate multisensory information in a united manner (Van Den Heuvel and Sporns, 2011; Harriger et al., 2012). These well-connected regions play a key role in global signal processing between different parts of the network, and functional disturbance of hub regions can have critical impact on overall network functioning. In this study we aimed to map the impact of transient functional brain disruption on global neural networks in the absence of structural damage in a newly developed reproducible rat model. To that aim, we characterized the brain's functional network organization with resting-state fMRI (rs-fMRI) after unilateral pentobarbital-induced brain “silencing.” We specifically focused on effects on well-connected hub regions using a relatively new framework that provides quantitative measures of the organization of highly connected rich-clubs (Van Den Heuvel and Sporns, 2011; Harriger et al., 2012; Collin et al., 2013). So far it has remained unknown whether rodent brain networks contain rich-club connections and if so, how these connections should be interpreted. Our hypothesis was that (i) rat brains have functional rich-club connections, and (ii) that transient hemispheric functional disruption affects the rich-club organization in both the disrupted and unaffected network regions.

## Methods

### Rat model

We developed a rat model of transient functional hemispheric disruption using unilateral focal anesthesia, similar to the intracarotid sodium amobarbital procedure for the “Wada test” in humans (Wada and Rasmussen, 2007). This procedure was chosen to temporally disrupt intra- and interhemispheric functional connectivity (FC) of the ipsilateral hemisphere. The protocol was approved by the Ethical Committee on Animal Experiments of the University Medical Center Utrecht and Utrecht University. Experiments were carried out in accordance with the guidelines of the European Communities Council Directive.

Three healthy adult Sprague-Dawley rats (Charles River Laboratories International Inc., MA, USA) were mechanically ventilated with 2% isoflurane in air: O_2_(7:1; rate 52–59 min^−1^). To catheterize the right carotid artery, the right common, and external carotid arteries were exposed by a ventral incision in the neck. A small catheter was advanced into the external carotid artery in the direction of the common carotid artery until the blunt catheter tip reached the bifurcation of the internal and external carotid arteries. Blood flow from the common into the internal carotid artery was ensured to be maintained (that is, the catheter tip remained in the ligated external common artery). The catheter was kept in place with a 5–0 suture and instant glue. The incision was closed and animals received 5 mL glucose-saline solution to compensate for loss of water and minerals.

Directly after surgery, rats were placed in an MR-compatible stereotactic holder and immobilized with earplugs and a tooth-holder. Blood oxygen saturation and heart rate were continuously monitored with a pulse-oximeter with the probe positioned on a hindpaw. In addition, expired CO_2_ was continuously monitored with a capnograph, and body temperature was maintained at 37.0 ± 0.5°C using a feedback-controlled heating pad. End-tidal CO_2_ levels were kept within the normal range, equivalent to arterial pCO_2_ levels between 35 and 45 mmHg (calculated from previous calibration measurements), by adjusting ventilation volume and rate when necessary.

### MRI

MRI experiments were done at 4.7 T with a 125 mm internal diameter gradient-coil insert. A Helmholtz volume coil and an inductively coupled surface coil were used for signal excitation and detection, respectively.

Repetitive blood oxygenation level-dependent rs-fMRI scans were acquired using a gradient echo single shot echo-planar imaging (EPI) sequence [repetition time (TR)/echo time (TE) = 500/19 ms; 7 1.5-mm coronal slices; field-of-view = 32 × 32 mm; in-plane voxel resolution, 0.5 × 0.5 mm; pulse angle = 35°; temporal scan resolution = 500 ms; total scan time = 15 min]. Exactly 10 min prior to rs-fMRI acquisition, end-tidal isoflurane concentration was reduced to 1.0%, which was maintained during rs-fMRI.

After 7.5 min rs-fMRI acquisition, 175 μL of 2 mg/kg pentobarbital was slowly injected intracarotidly. This concentration was based on an electroencephalography (EEG) titration/dose-response experiment, which showed that suppression of EEG activity occurred at this threshold level at 1% isoflurane anesthesia (data not shown). EEG activity returned to baseline within 15 min.

In one animal, perfusion MRI was conducted (TR/TE = 100/25 ms) following rs-fMRI, using gradient echo EPI (TR/TE = 100/25 ms; 11.5-mm coronal slice; field-of-view = 32 × 32 mm; in-plane voxel resolution, 0.5 × 0.5 mm; pulse angle = 90°) in combination with an bolus injection of 50 μL of 1.0 mmol/mL gadobutrol through the intracarotid catheter. Serial images were inspected to verify unilateral bolus arrival.

### EEG

To validate functional MRI measurements, EEG was recorded immediately following rs-fMRI acquisitions, at 1.0% isoflurane anesthesia outside the MR scanner. To that aim a small medial incision was made in the skin covering the skull and the pericranium. A hole in the skull above the left and right primary somatosensory cortices was made with a 300 μm micro drill. Next, epidural EEG was recorded from the bilateral somatosensory cortices with a reference electrode above the cerebellum. EEG activity was recorded for 15 min in two animals. After 7.5 min of EEG measurements, 175 μL of 2 mg/kg pentobarbital was slowly injected intracarotidly. The time between this injection and the first injection in the MR scanner was >1 h. EEG measurements were conducted using a homebuilt multichannel amplifier, and band-pass filtered between 0.1 Hz and 250 Hz, using a National Instruments™ NI USB-6211 DAQ with a sampling rate of 1000 Hz per channel.

### Image processing and analysis

Rs-fMRI images were motion-corrected, spatially smoothed, band-pass filtered between 0.01 and 0.1 Hz, and nonlinearly matched with the Paxinos and Watson rat brain atlas (Paxinos and Watson, 2006) for FC analysis. The first (prebarbital) and last (postbarbital) 500 images were used to extract time series for 32 regions-of-interest (ROIs) in each hemisphere, and to calculate pair-wise FCs between all ROIs from the correlation coefficient *r*. ROIs were carefully selected to basically include all bilateral neocortical and subcortical gray matter atlas regions (see Figure 2). The cerebellum was excluded from analysis. All ROIs were large enough to contain multiple voxels at the applied fMRI resolution, and were functionally connected to at least one other region.

### Estimation of weighted rich-club effect

Pre- and postbarbital FCs were averaged across animals to construct two 64 × 64 node networks (each node is a cortical or subcortical ROI in one of the hemispheres). From these networks ipsilateral and contralateral 32 × 32 node sub-networks were extracted. Negative FCs were excluded to allow calculations according to the weighted rich-club methodology (Opsahl et al., 2008).

The weighted rich-club effect characterizes the tendency of network hubs to engage in stronger or weaker FCs among them than expected by chance. The calculation involves multiple steps. First, all network nodes are ranked in terms of prominence. We used node strength *s* as a measure of prominence. Strength is defined as the sum of FCs attached to the connections originating from a network node (Barrat et al., 2004). For increasing values of *s*, we selected the group (the club) of all nodes whose strengths were larger than *s*. In this step we obtain a series of increasingly selective clubs. For each of these clubs we counted the number *E*_>*s*_ of connections connecting the club members, and measured the sum *W*_>*s*_ of the FCs attached to these connections.

In the next step we determined the ratio ϕ^*w*^(*s*) between *W*_>*s*_ and the sum of the FCs attached to the *E*_>*s*_ strongest connections within the network. Mathematically:

$$\phi^{w}(s)=\frac{W_{>s}}{\sum_{l\;=\;1}^{E_{>r}}w_{l}^{rank}}$$

where *w^rank^_l_* ≥ *w^rank^*_*l*+1_ with *l* = 1, 2, …, *E* the ranked FCs on the connections of the network. *E* is the total number of connections. With this formulation we determined the fractions of FCs shared by rich nodes compared with the total amount they could share if they were connected through the strongest connections of the network.

A high value of ϕ^*w*^(*s*) is not sufficient to account for the weighted rich-club effect as higher-strength nodes in a network have a higher probability of sharing connections with each other just by chance. In other words, networks where connections are randomly established could have a non-zero value of ϕ^*w*^(*s*). To control for this effect we normalized ϕ^*w*^(*s*) relative to the average weighted rich-club effect, ϕ^*w*^_random_(*s*), found in 1000 random networks generated using a reshuffle procedure. This procedure reshuffles the FCs globally in the networks, while keeping the topology intact.

The weighted rich-club effect ρ^*w*^(*s*) at strength *s* is the ratio: $\rho^{w}(s)\frac{\phi^{w}(s)}{\phi_{\text{random}}^{w}(s)}$. When ρ^*w*^(*s*) is > 1.0 the functional network has a positive weighted rich-club effect, with nodes in the rich-club concentrating most of their connections toward other club members relative to random networks.

### EEG analysis

EEG signals were analyzed in consecutive epochs of 30 s. For each epoch the correlation coefficients between the temporal fluctuations of the EEG signals from left and right somatosensory cortices were calculated for the delta (1–5 Hz), theta (5–8 Hz), alpha (8–15 Hz), beta (15–30 Hz), gamma (30–250 Hz), and broad (1–255 Hz) frequency bands.

## Results

**Figure 1:**
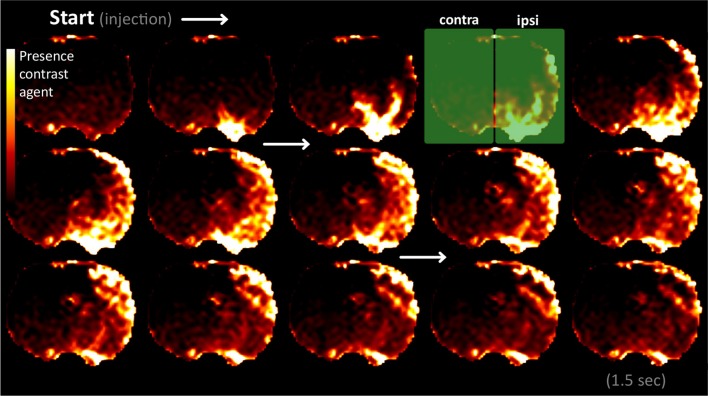
**Perfusion MRI frames, demonstrating clear unilateral delivery of the contrast agent in the hemisphere ipsilateral to the side of cannulation.** The frames were acquired at 100 ms intervals from the start of bolus administration until 1.5 s later (the temporal frame sequence runs from top left to bottom right). The frames are in coronal plane at 1 mm from bregma. Presence of contrast agent is displayed using a color scale: dark for low contrast concentrations and bright for high contrast concentration (in arbitrary units). The green rectangles mark the ipsi- and contralateral hemispheres.

Perfusion MRI data demonstrated that the catheterized carotid artery predominantly supplied the ipsilateral hemisphere allowing for selective hemispheric brain silencing with pentobarbital (Figure 1). Perfusion was strongest in the cortical areas, whereas levels in subcortical regions were lower. We also detected some contrast agent arrival in the anterior contralateral hemisphere, which can be explained by delivery via the circle of Willis.

### Network prominence

**Figure 2:**
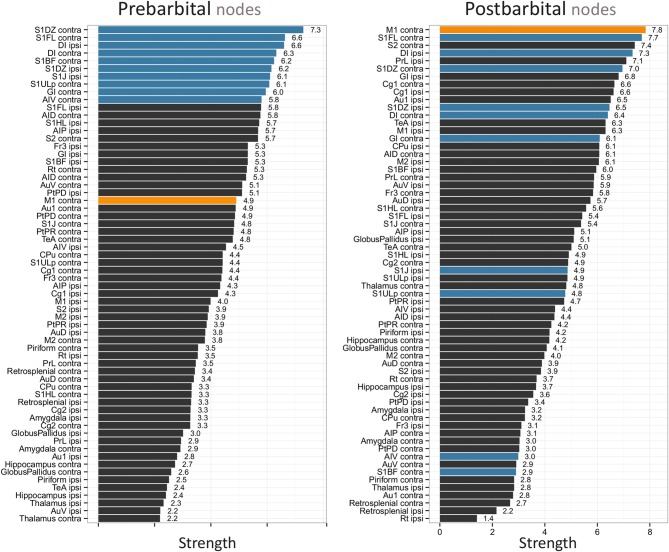
**Pre- and postbarbital individual network node strengths plotted as bar graphs.** The 64 bilateral nodes are sorted from high to low strength. The 10 network nodes with the highest strength prior to pentobarbital injection are given in blue. The node with the highest strength in the postbarbital functional network, the contralateral primary motor cortex, is given in orange. The following abbreviations are used, contra, left hemisphere, contralateral to cannulation, ipsi, right hemisphere, ipsilateral to cannulation; AID, agranular insular cortex dorsal part; AIP, agranular insular cortex posterior part; Au1, primary auditory cortex; AuD, secondary auditory cortex dorsal area; AuV, secondary auditory cortex ventral area; AIV, agranular insular cortex ventral part; Cg1, cingulate cortex area 1; Cg2, cingulate cortex area 2; CPu, caudate-putamen complex; DI, facial nucleus; Fr3, frontal cortex area 3; GI, granular insular cortex; M1, primary motor cortex; M2, secondary motor cortex; PrL, prelimbic cortex; PtPD, parietal cortex posterior area dorsal part; PtPR, parietal cortex posterior area rostral part; Rt, intermediate reticular nucleus; S1BF, primary somatosensory cortex barrel field; S1DZ, primary somatosensory cortex dysgranular zone; S1FL, primary somatosensory cortex forelimb region; S1HL, primary somatosensory cortex hindlimb region; S1J, primary somatosensory cortex jaw region; S1ULp, primary somatosensory cortex upper lip region; S2, secondary somatosensory cortex; TeA, temporal association cortex.

The prominence of all network nodes, measured as the strength, is plotted in Figure 2 for the prebarbital (baseline) and postbarbital whole-brain functional networks, as calculated from the rs-fMRI experiments. The ten most prominent prebarbital ROIs included mostly somatosensory regions, namely, the ipsi- and contralateral dysgranular zone of the primary somatosensory cortex; the contralateral forelimb region, upper lip region and barrel field of the primary somatosensory cortex; and the ipsilateral jaw region of the primary somatosensory cortex. The agranular and granular insular cortex and the facial nuclei were also prominent network nodes before pentobarbital injection.

Following unilateral pentobarbital injection, a subgroup of these ROIs lost prominence (Figure 2). This was most pronounced for the contralateral barrel field of the primary somatosensory cortex and the agranular insular cortex. In contrast, the prominence of the contralateral primary motor cortex increased toward the highest value in the postbarbital network. The ROIs presenting with the largest changes in prominence were not confined to a single anatomical region.

### Functional connectivity

**Figure 3:**
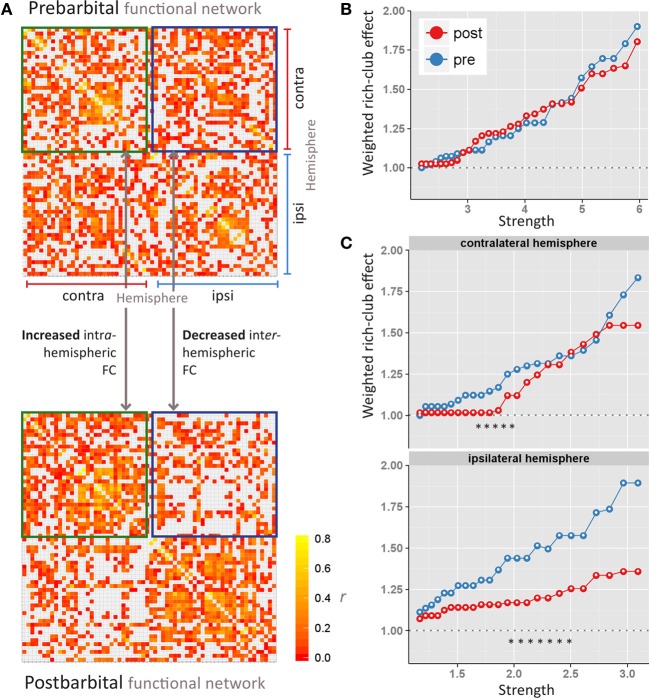
**Pre- and postbarbital (A) whole-brain functional connectivity (FC) networks, displayed as matrices.** Each row and column stands for a region-of-interest in the hemisphere ipsi- or contralateral to the side of cannulation. Matrix elements are colored according to FCs values, measured as the correlation coefficient *r* between regions-of-interest. Red indicates no FC; yellow indicates high FC. Interhemispheric FCs are delineated with blue squares; intrahemispheric FCs with green squares. Weighted rich-club effects (y-axis) are plotted against increasing levels of strength (x-axis) for the entire brain network **(B)**, and the contra- and ipsilateral **(C)** hemispheric networks. The maximum strength threshold depends on the size of the network. The strength thresholds were increased until networks started to disintegrate. This explains the different scaling of the x-axis in the hemispheric networks as compared to the entire brain network. Significant differences in weighted rich-club effects between prebarbital (blue line) and postbarbital (red line) networks are indicated as ^*^ [permutation analysis; *p* < 0.01 considered significant].

The average baseline functional network, i.e., before pentobarbital injection, displayed strong connectivity within and between hemispheres. After pentobarbital injection the ipsilateral hemisphere revealed increased intrahemispheric connections with concomitant decrease of interhemispheric FC (Figure 3A). Increased intrahemispheric FC was found in both hemispheres. Several ipsilateral regions retained strong network strengths with other regions, including the facial nucleus, prelimic cortex, cingulate cortex area 1, and the granular insular cortex.

### Weighted rich-club effect

The bilateral functional network was characterized by a strong weighted rich-club effect, with ρ^*w*^(*s*) > 1.0 over a range of prominence thresholds (Figure 3B). This rich-club effect was not affected by ipsilateral FC disruption. In contrast to the whole brain network, the ρ^*w*^(*s*) values were significantly decreased in the ipsilateral sub-network following unilateral pentobarbital injection (Figure 3C). This pattern of decreased ρ^*w*^(*s*) values was to a lesser extent also seen in the contralateral sub-network.

### Data variation

**Figure 4:**
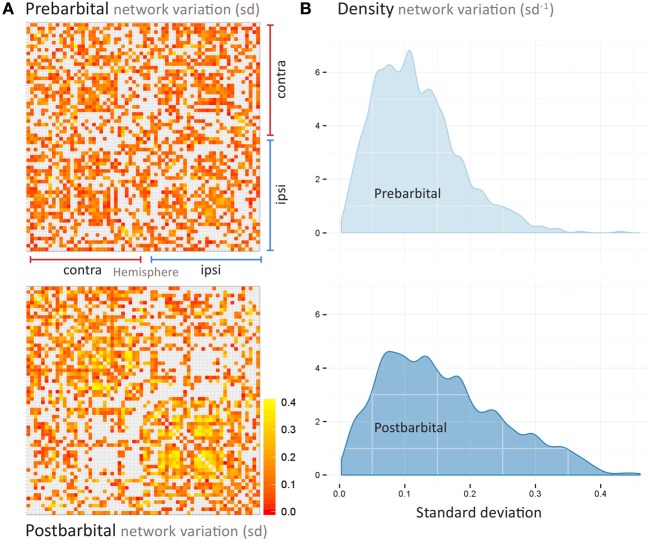
**Pre- and postbarbital (A) variation within the whole-brain functional connectivity matrices as shown in Figure 3.** Each row and column represent a region-of-interest in the hemisphere ipsi- or contralateral to the side of cannulation. Matrix elements are colored according to the standard deviation (*SD*) of the individual functional connectivity values, measured as the correlation coefficient *r* between regions-of-interest. Red indicates *SD* values close to 0; yellow indicates *SD* values close to 0.4. As shown in the density distribution of all *SD* values **(B)**, the variation between individual networks is slightly smaller for the prebarbital data (most *SD*'s between 0 and 0.3) as compared to the variation in the postbarbital data (with most *SD*'s between 0 and 0.4).

The variation between individual functional networks is shown in Figure 4. The standard deviation of the FC values slightly increased post pentobarbital injection as shown from the density plots. The standard deviation range was 0–0.3 prior to injection and 0–0.4 post injection.

### Electroencephalography

**Figure 5:**
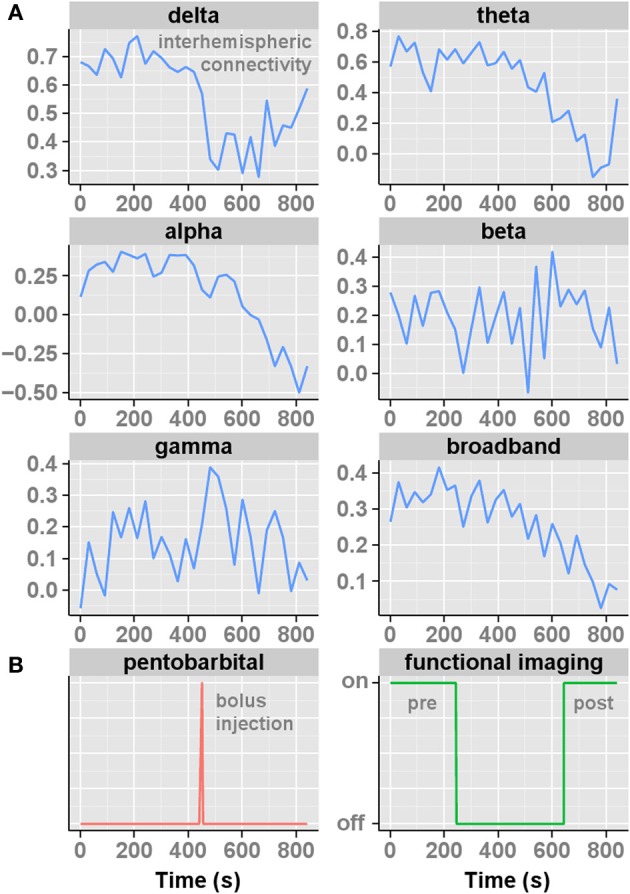
**The interhemispheric somatosensory functional EEG connectivities (measured as correlation coefficients (*r*)) for consecutive epochs, each 30 s, plotted over time (15 min) for the distinct frequency bands (A).** The corresponding moment of pentobarbital injection and periods of resting-state fMRI used to construct functional networks are shown below **(B)**. Note that functional imaging was acquired prior to EEG, but was identical in approach and timing of pentobarbital injection.

The EEG measurements, acquired in the same animals directly after imaging, supported our MRI-based observation of decreased interhemispheric resting-state FC. Interhemispheric EEG synchronization decreased after unilateral pentobarbital injection in the alpha, theta, and broadband frequencies (Figure 5). The synchronization in the other frequency bands increased temporarily (gamma) or remained unchanged (beta).

## Discussion

Our pilot study shows that transient functional global network disruptions in response to unilateral and reversible functional brain disturbance can be effectively studied using a straightforward rodent model in combination with rs-fMRI. Our rat data support the concept that densely connected rich-club regions play a central role in global brain communication, as has been previously shown in humans. In addition, we found that global network hub configurations can be significantly affected by temporary functional disruption of a hemisphere.

A rodent model that allows selective and reversible functional hemispheric silencing has not been described before. Our results show that this model can be useful to assess effects of (transient) brain impairment on functional networks. The concept of this model was based on recent FC and network data presented by Douw et al. (2009, 2010). They showed that FC within and between hemispheres changed in persons with epilepsy during an intra-arterial amobarbital injection—the WADA test—as measured with EEG (Douw et al., 2009). They concluded that the effects of unilateral anesthesia on FC in the contralateral hemisphere should not be ignored. Direct comparison of low-frequency rs-fMRI connectivities with the different EEG frequency bands is difficult, and more data are required to elucidate differences and similarities between rs-fMRI and EEG patterns. Nevertheless, the observed decrease in interhemispheric EEG FC in the delta and theta bands by Douw and colleagues corresponds with our interhemispheric EEG results (Figure 5). Over the ipsilateral hemisphere the delta and theta band synchronization increased after amobarbital injection in humans, which also agrees with our findings of increased ipsilateral intrahemispheric FC in rat brain after pentobarbital injection. Apparently, unilateral functional brain disruption by amo- or pentobarbital injections is confined to subverted functional outcome (that is, speech and language comprehension in humans) rather than absolute inhibition of low-frequency FC. This idea is further supported by recent FC measurements at an anesthetic depth characterized by unresponsiveness, where reductions in connectivity were shown to be partial and not complete (Hudetz, 2012).

Douw et al. also assessed EEG data acquired during WADA-test using whole-brain functional network analysis (Douw et al., 2010). Their results revealed an increased random network organization after unilateral functional disruption with amobarbital in people with epilepsy (Douw et al., 2010). However, the network topologies within the injected and non-injected hemispheres were not studied separately in this group of patients. In our study we found no difference in rich-club characteristics between pre- and postbarbital whole-brain networks. Differences in weighted rich-club effects were only found in sub-network analyses of individual hemispheres (Figure 3). The asymmetric presentation of prebarbital hub regions (that is, mostly contralateral sensorimotor areas) is in line with studies in healthy rats that reported right-sided population bias and functional lateralization (Glick and Ross, 1981; Alonso et al., 1991).

A positive weighted rich-club effect characterizes the tendency of network hubs to engage in stronger functional connectivities among them than expected by chance. We found significantly decreased ρ^*w*^(*s*) values in the ipsi- and contralateral sub-networks after unilateral pentobarbital injection. Similar positive rich-club organizations have been described at a neuronal level in the nematode worm *Caenorhabditis elegans* (Towlson et al., 2013) and at a larger scale in human neocortical networks (Van Den Heuvel and Sporns, 2011). Our findings in rodent brain networks, consolidate the hypothesis that connections according a rich-club layout may be a general and scale-invariant principle of brain network organization (Towlson et al., 2013). Nonetheless, further validation of this hypothesis is essential, given the small sample size used in this study.

The rich-club connections in the structural human brain network are considered to be the backbone for global brain communication (Van Den Heuvel et al., 2012). The majority of these connections are long-distance neural pathways, such that the rich-club phenomenon is costly in terms of consumed energy and space. However, the trade-off for higher cost is higher performance in terms of network integration; up to 70% of all network's shortest paths travel through rich-club connections.

Whether structural network principles also hold for functional rich-club networks remains to be determined, but we expect the functional rich-club organization to follow the underlying structural organization to a large extent. On the other hand, functional rich-club connections may be much more flexible in acutely responding to environmental changes, such as induced by anesthesia. This is, for instance, underscored by the differences in our whole-brain and sub-network data (Figures 3B,C). Unilateral brain silencing did not affect the rich-club effect at a whole-brain level; only the club members changed dramatically (Figure 2). On the other hand, the hemispheric sub-networks did reveal change in rich-club connectivity after hemispheric silencing. Apparently the level of network definition is critical for detecting changes in functional rich-club organization after functional brain lesioning. A better understanding of this phenomenon may require the acquisition of structural diffusion tensor imaging data alongside rs-fMRI in future studies. This will allow comparison of functional networks and their rich-club organization with data from the intact structural networks.

Limitations in this proof-of-principle methods study should be acknowledged. First, experiments were conducted at 1.0% isoflurane anesthesia. We do not know how this baseline anesthesia may have interacted with the pentobarbital-induced brain silencing. We expect this effect to be relatively small as cerebral glucose utilization, cerebral blood flow coupling (Maekawa et al., 1986; Lenz et al., 1998) and FC, including the long distance connections (Liang et al., 2012), are largely preserved at 1.0% isoflurane anesthesia (Wang et al., 2011). We also know that the neurovascular coupling in rat brain is similar across distinct types of anesthetics including pentobarbital and isoflurane (Franceschini et al., 2010), however further validation studies, including robust dose-response experiments, are needed. Rs-fMRI acquisition may be ideally executed in awake rats, however, this is complicated due to motion artifacts related to post-surgical pain, stress and discomfort.

Second, anesthesia-based functional brain disruption by unilateral injection through the carotid artery may not be exclusively unilateral due to presence of the circle of Willis and recirculation effects. Nonetheless, the small injection volume used in our study limited this effect, which was confirmed by the strongly lateralized results for hemispheric FCs.

Third, the likelihood that a statistically significant result reflects a true effect is reduced in studies with a small sample size (Button et al., 2013). The large changes in functional network connectivity and organization that we observed in this rat model alleviate this problem. Nonetheless, firm conclusions on directionality of network changes and size of effect require additional studies with larger groups of animals. This is emphasized by the variation that we found between FC correlation coefficients obtained from the individual networks (Figure 4). This variation is however comparable to the variation found between individual interhemispheric FC in rats recovering from a unilateral stroke, as studied with rs-fMRI with a similar experimental setup and design (Van Meer et al., 2012).

In conclusion, our pilot study suggests that changes in rich-club organization following functional network disruption might affect the capacity to efficiently segregate and integrate multisensory information. These changes are unlikely to depend on the presence of structural damage. We speculate that functional rich-club alterations may reflect an underlying mechanism of neurological impairments in patients with no or limited structural brain damage, such as after transient ischemic attack.

### Conflict of interest statement

The authors declare that the research was conducted in the absence of any commercial or financial relationships that could be construed as a potential conflict of interest.
